# Different predictors after stroke depending on functional dependency at discharge: a 5-year follow up study

**DOI:** 10.1186/s12883-020-01840-y

**Published:** 2020-07-01

**Authors:** Emma Westerlind, Daniel Hörsell, Hanna C. Persson

**Affiliations:** Institute of Neuroscience and Physiology, Department of Clinical Neuroscience, Sahlgrenska Academy, Sahlgrenska University Hospital, Per Dubbsgatan 14, 413 45 Gothenburg, Sweden

**Keywords:** Stroke, Stroke impact scale, Follow-up studies, Rehabilitation

## Abstract

**Background:**

Level of dependency after a stroke is important for long-term outcome in several aspects, but less is known about important predictors for outcome depending on functional dependency. The aim of the current study was to investigate self-perceived outcome and identify possible predictors of strength, participation, and emotional outcome 5 years after stroke based on functional dependency at discharge from hospital.

**Methods:**

This observational cohort study included participants living in Gothenburg that were diagnosed with first ever stroke in 2009 and 2010. Baseline data were gathered from medical charts and the outcome was based on the Stroke Impact Scale (SIS) questionnaire mailed out 5 years post-stroke. Logistic regression identified potential predictors of better strength, participation, and emotional outcome.

**Results:**

A total of 266 participants responded to the SIS. The functionally independent participants at hospital discharge reported significantly better scores in all SIS domains compared to the functionally dependent. For those who were functionally independent, only non-modifiable factors (age, sex, stroke type) were significant predictors of a better outcome. However, for the functionally dependent participants, modifiable factors such as feeling depressed, cardiovascular risk factors, and recurrent stroke were significant predictors of unfavourable outcome.

**Conclusions:**

Important factors for predicting a favourable outcome differed due to the level of functional dependency, and modifiable factors were only present in participants that were functionally dependent at discharge. Prevention, detection, and treatment of modifiable factors give an opportunity to reduce the burden of stroke for those who are most vulnerable.

## Background

Stroke is the second largest cause of death worldwide and the third largest cause of disability adjusted life years [1]. In high-income countries such as Sweden, mortality from stroke decrease, while the incidence of stroke increase in low and middle-income countries [2, 3]. This, in combination with an ageing and growing population, has resulted in a substantial increase in persons living with stroke [1, 4]. The consequences of a stroke are diverse and can depend on factors such as type of stroke, affected brain area, age, comorbidities and time to treatment [5–7]. The subsequent disability may not only be physical, but can also involve cognitive [8] and psychological problems [9]. Although most motor recovery after stroke seems to occur within the first 3 months [10], improved function and activity as well as improved self-perceived outcome has also been reported later [11]. However, a decline in self-perceived outcome within the first year has been shown [11, 12]. In the long-term perspective, there has also been reported a decline within 6 years [13], with the most affected areas being strength, hand function, and participation [13]. Concerning cognitive impairment, a previous study have shown a potential recovery between 3 months and 3 years post-stroke, even though cognitive impairment substantially occur at both time points [14].

Comorbidities such as atrial fibrillation, diabetes, and previous myocardial infarction of the person with stroke, has been shown to affect functional dependency [6]. More severe stroke [6] and older age [15] seem to additionally predict an unfavourable outcome after stroke. Being a woman has shown to be a risk factor for a higher degree of dependence, institutionalisation, depression, and unfavourable outcome post-stroke [16, 17]. Suggested explanations for this are that women generally are older and frailer at stroke onset [18], and that women have less support from their spouses and more often live alone [19]. Women seem to receive fewer diagnostic and therapeutic interventions than men after a stroke even when the risks and benefits of the interventions are the same [20]. Depression is common after stroke, nearly one out of four persons that had a previous stroke showed signs of depression in a population based study [21]. Post-stroke depressive symptoms, functional symptoms, functional independency, and lower health related quality of life are correlated to each other, indicating that treating depression is important after a stroke [19].

The initial stroke severity as well as age are strong predictors of long-term functional outcome [22, 23]. However, there is lack of knowledge regarding which specific factors, dependent on the initial functional dependency at discharge, that contribute to recovery. The aim of the study was to investigate self-perceived outcome, as well as identify possible predictors of strength, participation, and emotional outcome 5 years after stroke based on functional dependency at discharge from hospital.

## Methods

This is an observational cohort study with a 5-year follow-up post-stroke. The STROBE-guidelines for observational studies were followed. Data was extracted from the extended Stroke Arm Longitudinal study at the University of Gothenburg (SALGOT-extended) [24–26]. Participants resident in the Gothenburg urban area (within 35 km from the Sahlgrenska University Hospital), 18 years or older, admitted with a first time ischemic stroke (I63), intra cerebral haemorrhage (I61) or non-traumatic subarachnoid haemorrhage (I60) during 18 months in 2009–2010 at the neurosurgical clinic, stroke unit or the intensive care unit at the largest of the three hospitals creating Sahlgrenska University Hospital were eligible for inclusion in the present study. The Sahlgrenska University Hospital is the only centre in the area that provides interventions such as thrombectomy and thrombolysis.

Five years after stroke, the survivors received a questionnaire survey by mail. The survey included the Swedish version of the Stroke Impact Scale (SIS) 3.0 and the annual follow-up questionnaire from the Swedish Stroke Register. Only participants who replied to any of the SIS-questions were eligible for inclusion in the current study.

Baseline data from the acute phase were collected from medical charts. The National Institutes of Health Stroke Scale (NIHSS) [27] and the Hunt and Hess scale (H&H) [28] were assessed by medical doctor at hospital arrival. The NIHSS measures severity of neurological symptoms in ischemic stroke and intra cerebral haemorrhage and the scale range from 0 to 46, where a lower score is better. In the present study, a score of 0–2 was considered as very mild stroke, 3–4 as mild, 5–15 as moderate and > 16 as severe stroke. The H&H is used to assess severity of the subarachnoid haemorrhage. The H&H consists of five grades where a lower score suggests a less clinically severe presentation. The modified Rankin Scale (mRS) were assessed at discharge from hospital and was used to assess a person’s level of functional dependency. The mRS range from 0 to 5, and in the current study a dichotomisation of the mRS score was used where 0–2 represents functional independency and 3–5 functional dependency [29, 30]. Cardiovascular disease (CVD) was considered as cardiac arrhythmias, coronary artery disease, heart failure, heart valve disease, or septal defects. Cardiovascular risk factor (CVR) was considered as CVD, diabetes, hypertension, or hyperlipidaemia.

### Questionnaires follow-up

The current study includes two questions from the annual Swedish Stroke Register questionnaire. *Have you suffered a new stroke,* with the answers *yes* or *no.* The second question, *Do you feel depressed?* has five possible answers which was dichotomised as followed: *never/almost never/sometimes* corresponding to not feeling depressed and *often/all the time* as feeling depressed [31].

The SIS questionnaire [32, 33] captures different aspects of stroke outcome and consists of 59 items in eight domains with questions rated in an ordinal scale 1–5 covering: strength, memory and thinking, mood and emotion control, communication and language, activities of daily living (ADL), mobility, hand function, and participation. Each domain score were then transformed using the following equation: domain score = (mean item score-1)/5-1 × 100, with each domain receiving a final total score out of 100 [32, 33]. The composite physical domain (including strength, hand function, mobility and ADL) were used [32, 33]. If a participant responded to less than 50% of the items in a domain, the results from the domain were excluded. The SIS-domain scores were dichotomised in the regression analysis, with scores ≥80 considered as full function [34]. The SIS includes in addition a visual analogue scale (VAS) estimates the respondents self-perceived recovery from 1 to 100, a higher score is better.

### Statistical methods

All analyses were performed using IBM SPSS version 25.0. For analysing group differences, Mann–Whitney *U* test and Fischer’s exact test were used. Significance level was set to *p* < 0.05, two-tailed tests were used.

Based on the level of functional dependency (mRS), logistic regression analysis was used to; predict favourable outcome in each of the dichotomised SIS-domains: emotion, participation and composite physical. Potential predictor variables in each of the models were: recurrent stroke, feeling depressed, sex, age, stroke type, and CVR. Predictor variables were excluded prior to the multivariable analysis if: too few participants (< 5) in each subgroup, correlated rho > 0.7 or < − 0.7 to other predictors, or if the contribution of the predictor variables were non-significant (*p*-value ≥0.25) in univariable regressions. Goodness of fit and accuracy of the multivariable regressions were tested using the Hosmer and Lemeshow test, the Nagelkerke R^2^, and a Receiver Operating Characteristic (ROC)-curve. An area under the curve (AUC) of > 0.7 was considered acceptable accuracy, > 0.8 as excellent and > 0.9 as outstanding.

## Results

**Fig. 1:**
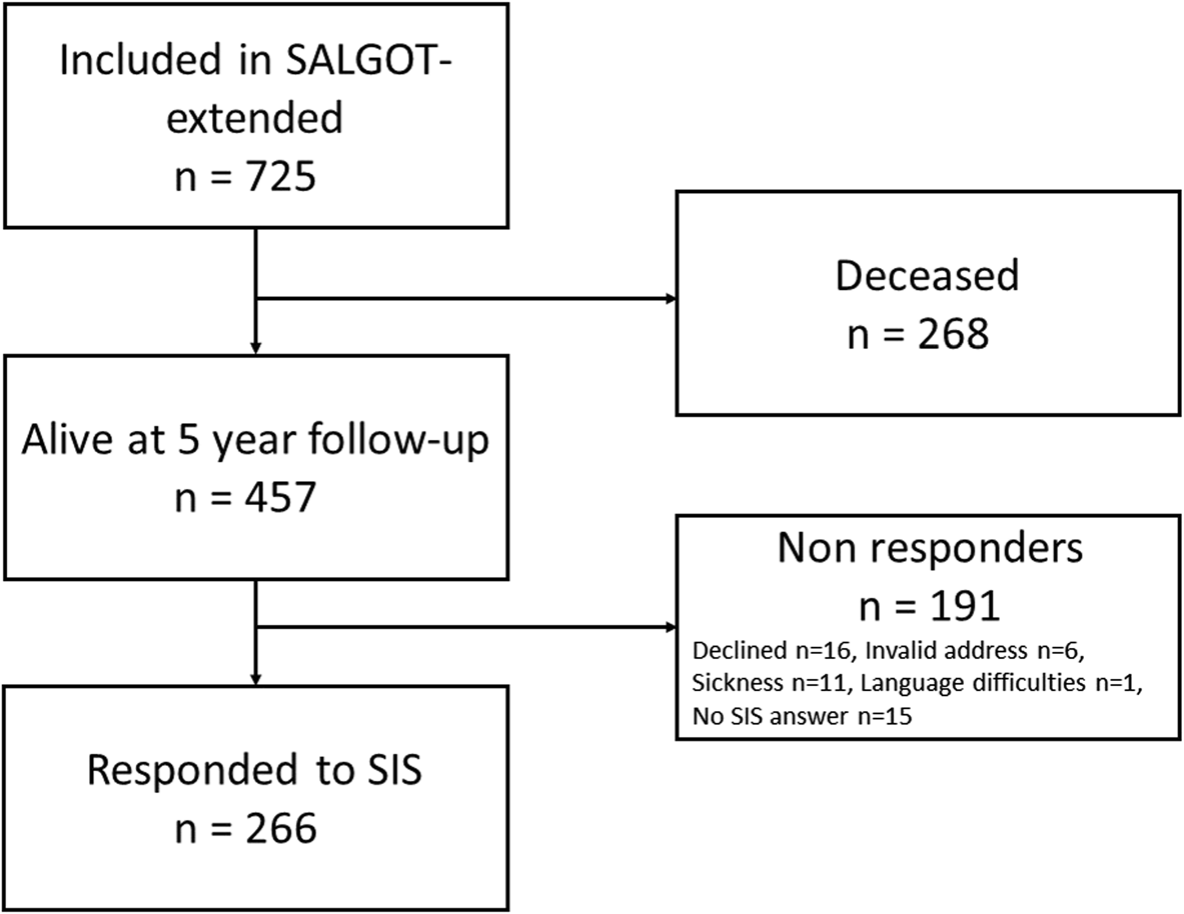
Flowchart of the inclusion process

Of the 457 persons that were alive 5 years post stroke, 266 persons (58%) responded to the SIS questionnaire in the follow-up survey (Fig. 1). There were no significant differences regarding age (*p* = 0.397), functional dependency at discharge from hospital (*p* = 0.145), aphasia at stroke onset (*p* = 1.0) or side of lesion (*p* = 0.107) between the responders (*n* = 266) and the non-responders (*n* = 191) to the follow-up questionnaires. However, there were significantly more females in the non-responders group (non-responders female *n* = 103 (53%), responders female *n* = 101 (38%), *p* = 0.001). As well as non-responders had longer length of stay in the hospital (mean (SD) 11.1 (13.78) compared to, 8.3 (8.03) *p* = 0.0005).

The majority of the participants were men, the mean age at the time of stroke was 65 years and most of the participants had a very mild stroke (Table 1). About half of the participants were functionally independent at discharge from hospital (Table 1). The participants that were functionally dependent at hospital discharge had a more severe stroke at onset (NIHSS and H&H). At 5 year post stroke, participants that were functionally dependent at discharge, reported a higher prevalence of feeling of depressed, 19% (25/136), compared to 9% (11/130) in participants that were functionally independent (Table 1).

**Table 1:** Demographic and clinical characteristics at baseline and 5 years post-stroke, divided according to functional dependency at discharge from hospital

	All participants	Functionally independent	Functionally dependent
Total, n (%)	266 (100)	130 (49)	136 (51)
**Baseline**			
Age, mean (SD)	65 (14)	64 (13)	66 (14)
Sex, n (%)			
Male	163 (61)	85 (65)	78 (57)
Female	103 (39)	45 (35)	58 (43)
Stroke type, n (%)			
Ischemic stroke	205 (77)	110 (85)	95 (70)
Intra cerebral heamorrhage	34 (13)	7 (5)	27 (20)
Subarachnoid heamorrhage	27 (10)	13 (10)	14 (10)
Side of lesion^a^, n (%)			
Right hemisphere	95 (39)	46 (39)	49 (39)
Left hemisphere	123 (51)	57 (49)	66 (53)
Bilateral lesion	7 (3)	3 (3)	4 (3)
Cerebellum	4 (2)	0 (0)	4 (3)
Unknown	13 (5)	11 (9)	2 (2)
NIHSS^b^ *, median (min-max)	1 (0–24)	1 (0–20)	2 (0–24)
H&H^c^, median (min-max*)*	2 (1–5)	2 (1–4)	2 (1–5)
Length of hospital stay^d^, mean (SD)	8 (7)	5 (4)	11 (8)
Comorbidity^e^, n (%)			
CVD	69 (26)	31 (24)	38 (29)
CVR	157 (60)	54 (42)	82 (62)
**Five years post-stroke**			
Recurrent stroke, n (%)			
Yes	41 (15)	21 (16)	20 (15)
No	225 (85)	109 (84)	116 (85)
Feeling depressed^f^, n (%)			
Never or almost never/Sometimes	221 (86)	116 (91)	105 (81)
Often/always	36 (14)	11 (9)	25 (19)

*Abbreviations*: *SD* Standard deviation, *NHISS* National Institutes of Health Stroke Scale, *H&H* Hunt and Hess, *CVD* Cardiovascular disease, *CVR* Cardiovascular risk

^a^*n* = 242. ^b^*n* = 215. ^c^*n* = 25. ^d^*n* = 237 ^e^*n* = 262. ^f^*n* = 257

At 5 years post stroke (mean 4,9 SD 0.50), the participants reported a median of 88–95 in all of the domains of SIS except for the strength domain (median 75) and the emotion domain (median 69) (Table 2). The participants that were functionally independent at discharge from hospital reported significantly higher scores in all domains of the SIS compared to participants that were functionally dependent (Table 2). The emotion domain had the lowest scores in both groups. Furthermore, self-perceived recovery according to the VAS (SIS), was higher among the functionally independent participants (median 90) compared with the functionally dependent participants (median 70).

**Table 2:** Outcome and recovery in each SIS domain, in all participants, and depending on functionally dependency

Domain	All participants, median (min-max)	Functionally independent, median (min-max)	Functionally dependent, median (min-max)	***P***-value
**Strength**, *n* = 234	75 (0–100)	100 (25–100)	69 (0–100)	**< 0.001**
**Hand function**, *n* = 255	95 (0–100)	100 (10–100)	75 (0–100)	**< 0.001**
**Mobility**, *n* = 263	92 (0–100)	100 (38–100)	86 (0–100)	**< 0.001**
**ADL/IADL**, *n* = 265	95 (0–100)	100 (23–100)	85 (0–100)	**< 0.001**
**Physical**, *n* = 222	92 (0–100)	98 (35–100)	82 (0–100)	**< 0.001**
**Emotion**, *n* = 262	69 (17–100)	75 (28–100)	67 (17–100)	**< 0.001**
**Language and communication**, *n* = 263	93 (11–100)	96 (39–100)	89 (11–100)	**< 0.001**
**Participation**, *n* = 258	88 (0–100)	97 (22–100)	78 (0–100)	**< 0.001**
**Memory**, *n* = 255	89 (11–100)	93 (32–100)	79 (11–100)	**< 0.001**

*Abbreviations*: *ADL* Activities of daily living, *IADL* Instrumental activities of daily living

Among the functionally independent participants, higher age was a significant predictor for lower odds of having favourable outcome in the emotion domain (OR 0.938) and the physical domain (OR 0.847), Table 3. In the participation domain however, only sex was significant with an OR of 0.351 for females to have a favourable outcome. Stroke type was a significant predictor of emotional outcome, with lower odds of favourable outcome for persons with haemorrage compared to ischaemic stroke (OR 0.251). The emotion and physical domain regression models had an acceptable and excellent accuracy (ROC-curve), however this was not seen in the participation domain model.

**Table 3:** Multivariable regression of predictors for favourable outcome and recovery in the emotion, physical and participation domain in the SIS questionnaire, for participants that were functionally independent at hospital discharge (mRS 0–2)

Functionally independent	Emotion		Physical		Participation	
	OR (95% CI)	*p*-value	OR (95% CI)	*p*-value	OR (95% CI)	*p*-value)
Stroke type, heamorrhage	0.251 (0.076–0.828)	**0.023**				
Age	0.938 (0.907–0.971)	**< 0.001**	0.847 (0.772–0.929)	**< 0.001**	0.978 (0.949–1.009)	0.158
Sex, female	0.563 (0.244–1.298)	0.178	0.452 (0.114–1.783)	0.257	0.351 (0.156–0.792)	**0.012**
Recurrent stroke					0.454 (0.163–1.264)	0.131
Nagelkerke R^2^	0.198		0.411		0.104	
ROC-curve, AUC	0.730		0.884		0.666	
Hosmer and Lemeshow test	0.658		0.997		0.480	

*Abbreviations*: *OR* Odds ratio, *CI* Confidence interval, *ROC* Receiver operating characteristics, *AUC* Area under the curve

As seen in Table 4, for participants that were functionally dependent at discharge, no significant predictor could be found for outcome in the emotion domain. In the physical domain, being female (OR 0.290), having a recurrent stroke (OR 0.257), feeling depressed (OR 0.347), and having CVR (OR 0.347) were all predictors for lower odds of having favourable outcome. In the participation domain, feeling depressed could predict outcome with an OR of 0.227. The physical regression model had an acceptable accuracy.

**Table 4:** Multivariable regression of predictors for favourable outcome and recovery in the emotion, physical and participation domain in the SIS questionnaire, for participants that were functionally dependent at discharge from hospital (mRS 3–5)

Functionally dependent	Emotion		Physical		Participation	
	OR (95% CI)	*p*-value	OR (95% CI)	*p*-value	OR (95% CI)	*p*-value)
*Age*	0.988 (0.958–1.018)	0.422	0.977 (0.941–1.015)	0.229	0.975 (0.942–1.009)	0.151
*Sex, female*	0.408 (0.158–1.058)	0.065	0.290 (0.115–0.728)	**0.008**	0.670 (0.298–1.505)	0.332
*Recurrent stroke*			0.257 (0.071–0.922)	**0.037**	0.383 (0.117–1.252)	0.112
*Feeling depressed*			0.311 (0.102–0.954)	**0.041**	0.227 (0.078–0.658)	**0.006**
*CVR*			0.347 (0.126–0.956)	**0.041**	0.817 (0.319–2.093)	0.673
Nagelkerke R^2^	0.059		0.297		0.173	
ROC-curve, AUC	0.630		0.756		0.695	
Hosmer and Lemeshow test	0.473		0.363		0.636	

*Abbreviations*: *OR* Odds ratio, *CI* Confidence interval, *ROC* Receiver operating characteristics, *AUC* Area under the curve

## Discussion

As seen in the present study, the level of dependency at discharge from hospital is still of importance in self-perceived outcome 5 years after a stroke. Participants who were functionally independent at discharge from hospital reported significantly more favourable outcome compared to functionally dependent participants. Different factors were important for predicting outcome in the emotional, physical and participation domains, depending on level of functional dependency at discharge from hospital. These new findings highlight the importance of considering the level of functional dependency early after stroke, to be able to give tailored rehabilitation.

Overall, the significant predictor for outcome in the functionally independent participants were all non-modifiable factors, including sex, age, and stroke type. In the functionally dependent participants, the predictors mainly included modifiable factors, such as recurrent stroke, feeling of depression, and CVR. This indicates that in the functionally dependent participants, it is important to detect, prevent and treat these factors in order to reduce the long term stroke burden. For participants that were functionally dependent at discharge, feeling depressed was a predictor in the physical domain, and the only contributing predictor in the participation domain. Depression has been shown to have a significant relationship to functional outcome after stroke [35] and depression itself is an important risk factor for stroke [36]. Since depression is an at least partly treatable condition, it is important for health care professionals to be aware of its importance for outcome after stroke, so that that the detection and treatment of depression can be optimised.

The CVR and recurrent stroke were significant predictors for outcome in participants that were functionally dependent. The CVR includes heart disease and diabetes, which also could be risk factors for functional restriction. Like myocardial infarction, multiple risk factors combined could cause an acute stroke [37]. Ten potentially modifiable risk factors are associated with 90% of stroke including untreated hypertension, psychosocial factors, obesity, tobacco smoking, heavy alcohol use, diabetes mellitus, inactive lifestyle, and unhealthy diet [37]. Increased physical activity has both physical and psychological benefits post stroke [38] and regular physical activity prevent a large number of risk factors for cardiovascular diseases [37]. In the present study, participants that were functionally dependent at discharge from hospital should be a specific target of preventive measures such as increased physical activity and use of medication for treatable conditions. The non-modifiable predictors, predominantly age and sex, were of importance for predicting outcome. Being female was a predictor of lower odds of favourable outcome in one of the physical models and one of the participation models. Generally women have less muscle mass to compensate deficits, women are generally older at stroke onset as well, which could partly explain the sex differences [39].

Participants had an overall good outcome in all of the SIS domains in the present study, regardless of functional dependency, and lowest scores were present in the emotion domain. Perceived emotion has been shown to be associated with perceived participation [34], and in the present study self-perceived participation was lower in participants that were functionally dependent, also seen previously [13, 40, 41]. It could be hypothesised that increased level of participation after stroke also could improve self-perceived outcome.

### Limitations

The present study has limitations that need to be discussed. The use of SIS gave information about self-perceived outcome after stroke in several aspects of health. The present study did however not contain any diagnostic assessments of for instance depression and anxiety or any information about medication for depression or anxiety, which limits the results. Furthermore, there is missing information about received rehabilitation after stroke, which could affect the results.

Other factors may be of importance for the regression models than the predictors included, indicated by the small AUC of the ROC-curves and pseudo-R^2^ in some of the regression models. For instance, the study does not have information on the socioeconomic background, educational level, type of work or comorbidities of the participants. The low goodness of fit in some of the models affects the strength of the model, and the results needs to be interpreted with caution.

The relatively low response rate of 58% could entail a risk of selection bias of the study population. The participants in the present study were more likely to have a subarachnoid haemorrhage, were younger, more often male and had less severe stroke than the general stroke population in Sweden [42]. This could be explained by old and frail patients not surviving the five-year follow-up to the same extent as younger patients. There were also a lower mortality rate among the people eligible for inclusion in the present study compared to the general Swedish stroke population [43]. Furthermore, the study was performed in an urban setting and at a university hospital with accessibility to interventions such as revascularisation and neurosurgery. This could cause selection bias and needs to be taken into account when generalising the findings.

## Conclusion

Five years post-stroke participants that were functionally independent at discharge had better outcomes in all domains compared to the functionally dependent. Depending on functional dependency at discharge, different predictors were important for a favourable outcome. Functionally independent participants only had non-modifiable predictors, whereas the functionally dependent participants mainly had modifiable predictors. This new information provides an opportunity to improve the situation for the most vulnerable stroke survivors, by providing tailored rehabilitation efforts, as well as preventing and treating risk factors.

## Data Availability

The data sets analysed during the current study are not publicly available due to ethical restrictions. According to the Swedish regulation http://www.epn.se/en/start/regulations/, the permission to use data is only for what has been applied for and then approved by the Ethical board. Data are available from the authors (contact Dr. Hanna C. Persson, email: hanna.persson@neuro.gu.se) upon reasonable request.

## Abbreviations

ADL: Activities of daily living
AUC: Area under the curve
CVD: Cardiovascular disease
CVR: Cardiovascular risk factor
H&H: Hunt & Hess
mRS: Modified Rankin Scale
NIHSS: National Institutes of Health Stroke Scale
OR: Odds ratio
ROC: Receiver Operating Characteristic
SALGOT: Stroke Arm Longitudinal study at the University of Gothenburg
SIS: Stroke Impact Scale
VAS: Visual analogue scale
